# Cortisol as an Independent Predictor of Unfavorable Outcomes in Hospitalized COVID-19 Patients

**DOI:** 10.3390/biomedicines10071527

**Published:** 2022-06-28

**Authors:** Renata Świątkowska-Stodulska, Agata Berlińska, Ewelina Puchalska-Reglińska

**Affiliations:** 1 Department of Endocrinology and Internal Medicine, Faculty of Medicine, Medical University of Gdańsk, Smoluchowskiego 17, 80-214 Gdańsk, Poland; renata.swiatkowska-stodulska@gumed.edu.pl; 2 Dialysis Unit, 7 Navy Hospital in Gdańsk, Polanki 117, 80-305 Gdańsk, Poland; e.puchalska@7szmw.pl

**Keywords:** cortisol, adrenal glands, COVID-19, SARS-CoV-2, interleukin-6

## Abstract

Most cases of COVID-19 are non-severe, but some patients require urgent hospital care. In the past, it has been established that adrenal hyperactivity predicts poorer prognosis in severely ill patients. We wanted to verify if cortisol levels can be tied to clinical outcomes and the degree of inflammation in hospitalized COVID-19 patients. We recruited 180 adult patients with PCR-confirmed COVID-19. The group was divided into smaller subgroups based on the glucocorticoid treatment status; the subgroups were evaluated in three separate time points. The assessment involved hormonal function (cortisol, ACTH), inflammatory markers, and occurrence of the pre-selected endpoints (death, hospitalization ≥10 days, non-invasive ventilation or high-flow oxygenation, mechanical ventilation, vasopressors). In the evaluated group, 121 patients showed signs of abnormal adrenal function. There was a clear correlation between cortisol and IL-6 concentrations in all three time points regardless of glucocorticoid treatment. A total of 71.1% of patients displaying abnormal cortisol production met the preselected endpoints. Our analysis showed that a cutoff cortisol concentration prognosing endpoint occurrence could be set at 15.45 μg/dL for patients not treated with glucocorticoids. Cortisol concentration can be seen as an independent prognostic factor for unfavorable outcomes in selected adults hospitalized with COVID-19.

## 1. Introduction

In March 2020, when the WHO declared COVID-19 a pandemic, little did we know about the global consequences of the rapid spread of the disease. Waves of new COVID-19 cases overburdened medical systems worldwide and up to this day, despite the wide availability of vaccination, no stabilization was reached. Well-known risk factors for poor prognosis include obesity, diabetes, arterial hypertension, immune deficiency, and old age. In up-to-date data provided by the WHO, nearly 6 million deaths from COVID-19 have been reported globally [1]. Over the course of the pandemic, scientists were able to track COVID-19-related abnormalities in many systems but only a few studies covering endocrine findings were published. Therefore, important data covering endocrine trends and prospective prognostic factors might be missing. It has been well-established over the past years that hormonal function tends to undergo substantial changes during acute and prolonged severe illness. For instance, low circulating free triiodothyronine/free thyroxine or excessive cortisol bursts are associated with higher mortality. In our study, we focused on adrenal function in adults hospitalized due to COVID-19. We wanted to check if it is possible to predict unfavorable outcomes as early as on day one of hospitalization and if any particular concentration of cortisol displays a prognostic importance.

## 2. Materials and Methods

We conducted a prospective single-center cross-sectional study aimed at investigation of hormonal parameters as possible prognostic factors in COVID-19.

We recruited 180 adult patients (≥18 years old) with PCR-confirmed COVID-19. The recruitment was open between 14 February 2021 and 1 December 2021, with the 7 Navy Hospital in Gdańsk as the study site. The study was conducted according to the Declaration of Helsinki and it was approved by the Independent Bioethics Committee for Scientific Research at Medical University of Gdańsk, Gdańsk, Poland (permissions NKBBN/373/2020, NKBBN/373-96/2021, NKBBN/373-184/2021). Written consent of patients or their legal guardians was obtained by recruiters. In some cases, patients were unable to give written consent due to their poor condition caused by COVID-19 and/or comorbidities. No patients withdrew their consent. There were no exclusion criteria other than the patient’s dissent to participation. The study was registered at ClinicalTrials.gov (NCT05070091).

To prevent potential sources of bias, we aimed at a protocol-based, unified approach in all of our patients. We collected anthropometric and metrical data, basic medical history, and history of medication use. Prospectively, we assessed vital signs, oxygen demand, use of steroids, critical events (mechanical ventilation, non-invasive ventilation (NIV/CPAP)/high-flow nasal oxygenation (HFNO), use of vasopressors, extended hospital stay (10 days or longer), death), and laboratory tests (adrenocorticotropin (ACTH), cortisol, C-reactive protein (CRP), interleukin 6 (IL-6), leukocytes, neutrocytes, lymphocytes).

Venous blood for laboratory tests was collected in the morning (6 AM–8 AM) by qualified medical personnel. We arranged blood collection on three separate occasions (days 1, 4, and 10 post-admission, later referred to as “time points”, with the main aim to provide a structured and repeatable frame for the study), with a 24 h time frame change allowed whenever special circumstances demanded it. The parameters assessed on all three days included ACTH, cortisol, CRP, IL-6, leukocytes, neutrocytes, and lymphocytes. All analyses were carried out in commercial laboratories. Additional information regarding laboratory assays is available as Table S1.

**Figure 1:**
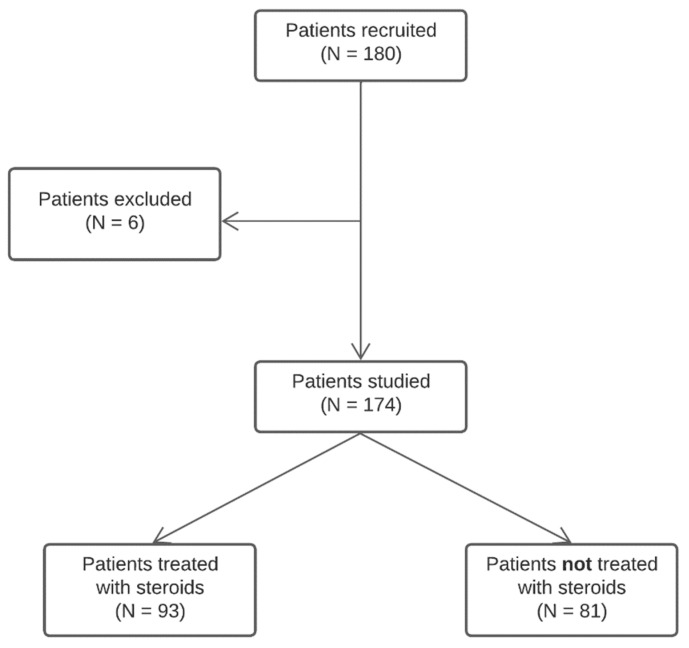
The history of group recruitment.

We excluded 6 patients from the final analysis due to inadequate clinical data coverage. As treatment guidelines for COVID-19 at the time of recruitment involved glucocorticoid (GC) administration, which can greatly influence the hypothalamus–pituitary–adrenal (HPA) axis, we divided the remaining 174 patients into two smaller subgroups: the treated with glucocorticoids group (GCG, N = 93) receiving dexamethasone or methylprednisolone, and the treated with no glucocorticoids group (NGCG, N = 81). The general definition for GCG included oral and/or parenteral steroid use at any point during hospitalization; at least one day of GC use prior to blood collection was required to be included in the GCG. The history of group recruitment is shown in Figure 1.

Statistical analysis was performed at the Centre of Biostatistics and Bioinformatics Analysis, the Medical University of Gdańsk, Gdańsk, Poland. The analysis was divided in two stages. First, statistical analysis of the whole cohort was performed.

The distribution of quantitative data was assessed using the Shapiro–Wilk W test. Quantitative data, based on the established distribution, were displayed as either median and interquartile range (if distribution deviated from normal distribution) or as arithmetic mean with standard deviation (if distribution was normal). Qualitative data were presented in absolute numbers and percentages.

Correlations between variables were evaluated using Spearman test for rank correlation. The strength of correlations was assessed by means of the Spearman r_SP_ correlation coefficient; the proportion of variation explained by correlation was estimated using the coefficient of determination R^2^. Differences between compared groups were tested using the Mann–Whitney U test.

Cortisol and ACTH changes over time were analyzed using repeated measures ANOVA (rmAOV); the rmAOV model involved time, GC use, and their interaction as main effects, with age, sex, and BMI as confounders. Analysis assumptions were checked by Mauchly’s sphericity test. If the sphericity assumption was not met, the significance level of time as the main factor was adjusted using Greenhouse–Geisser correction. Post-hoc multiple comparisons were carried out using the Student’s t test with FDR correction according to Benjamini and Hochberg whenever necessary.

Then, the usefulness of baseline cortisol level as the predictor of the COVID outcome was assessed by means of the combined logistic regression modelling and ROC curve analysis-based approach. To this end, the cohort was divided into the training set and the test set. The independent test set consisted of 20% of the patients selected randomly. GCG and NGCG groups were considered separately. 5 GCG and 5 NGCG patients were excluded from the analysis due to incomplete laboratory results. Two logistic regression models were analyzed: the first one considered just the baseline cortisol level, while the second one included also the patients’ age, sex and BMI as the confounders. An additional 14 GCG and 20 NGCG patients were excluded from the second model since they lacked the respective BMI data; 1 more patient was excluded in the prior step of the analysis (lacked both the laboratory results and BMI value). The performance of both models was assessed on the training set with the optimal model cut-off level being determined from the ROC curves based on the Youden index. Obtained models were then tested against the independent test set. Predictive ability of a model was evaluated using statistical measures including the area under the curve (AUC), sensitivity, specificity, and odds ratio (OR) with respective 95% confidence intervals (95% CI).

Statistical significance threshold was set at *p* < 0.05 for all analyses. All analyses were performed in the R environment.

## 3. Results

In general, the age of recruited individuals ranged from 18 to 94 years, and 84 patients (48.3%) were female. A total of 87 patients (50.0%) were hospitalized for at least 10 days. No patients had a known history of adrenal disorders, but 7 patients (4.0%) used steroids as medications for other conditions (3/7—methylprednisolone, 4/7—dexamethasone); in all these cases steroid treatment was continued throughout the hospital stay. History of chronic heart failure was reported in 17 (9.77%) patients, atrial fibrillation in 23 (13.22%), coronary artery disease in 30 (17.24%), stroke in 11 (6.32%), pulmonary embolism in 3 (1.72%), chronic obstructive pulmonary disease or asthma in 18 (10.34%), active neoplasia in 14 (8.05%), diabetes mellitus in 53 (30.46%), arterial hypertension in 102 (58.62%), chronic kidney disease in 26 (14.94%), and 17 (9.77%) patients required hemodialysis. We gathered information about BMI of 139 analyzed patients: mean BMI was 27.574 ± 5.251 kg/m^2^, 53 (38.1%) patients were overweight, and 39 (28.1%) were obese. A total of 100 out of 173 subjects (57.8%) needed oxygen support at one time point at least of their hospitalization. A total of 30 patients (17.2%) succumbed to their illness, 12 (6.9%) required NIV/CPAP/HFNO, 3 (1.7%) mechanical ventilation, and 6 (3.5%) vasopressor use. Characteristics of the studied population throughout the three time points and including steroid use or its lack are shown in Table 1, Table 2 and Table 3.

The GCG and the NGCG were compared with special regard for laboratory results and exogenous steroid use. We used cortisol as the primary parameter for assessment of adrenal function, as ACTH has secondary meaning in the HPA. Due to use of GCs in one of the groups, we decided on different thresholds for the NGCG and the GCG. For the NGCG, the cut-off values for abnormal adrenal function were set below 6.02 μg/dL (lower normal limit for the testing method) and above 18.4 μg/dL (upper normal limit for the testing method). For the GCG, the cut-offs were partially based on expected normal results of suppression tests: below 1.8 μg/dL (full suppression of cortisol secretion), below 5.0 μg/dL but above 1.8 μg/dL (partial suppression of cortisol secretion), paired with an additional cut-off set at 18.4 μg/dL (upper normal limit for the testing method). For both the NGCG and the GCG, we distinguished an alarmingly high concentration of cortisol which exceeded 36.2 μg/dL. Using the aforementioned criteria, we observed abnormal levels of cortisol in 121 patients: 70 from the GCG and 51 from the NGCG. Among patients displaying cortisol production abnormalities (N = 121), mean age was 69.033 ± 13.981 years, mean BMI was 28.068 ± 5.381 kg/m^2^, and 62 (51.2%) of these patients were female.

We studied endpoint completion in patients displaying and not displaying abnormal cortisol production. We defined our combined primary endpoint as occurrence of death, hospitalization period of 10 days or longer, use of NIV/CPAP/HFNO, use of mechanical ventilation, and/or use of vasopressors. Secondary endpoints were met if any of the listed above events occurred. A total of 86 out of 121 patients (71.1%) with abnormal adrenal function met the preselected endpoints; 69/121 (57.0%) were hospitalized for at least 10 days (mean duration of hospitalization: 13.347 ± 8.924 days), 24/121 (19.8%) died, 12/121 (9.9%) required non-invasive ventilation, 1/121 (0.8%) mechanical ventilation, and 4/121 (3.3%) use of vasopressors. A total of 21 out of 53 (39.6%) patients with normal adrenal function met the preselected endpoints: 18/53 (34.0%) were hospitalized for at least 10 days (mean duration of hospitalization: 9.058 ± 6.013 days), 6/53 (11.3%) died, 2/53 (3.8%) needed mechanical ventilation, 2/53 (3.8%) use of vasopressors, and no patients needed non-invasive ventilation.

We assessed cortisol levels in the GCG and the NGCG in relation to pre-set thresholds. Some patients displayed different types of laboratory abnormalities depending on the time point of the assessment. In the GCG, full suppression of cortisol production in at least one of the three time points was seen in 46 subjects (49.46%), partial suppression in 36 (38.71%), cortisol concentration exceeding the upper limit of the laboratory norm in 29 (31.18%), and no patients exceeded the cortisol level of 36.2 μg/dL. In the NGCG, 8 (9.88%) patients displayed cortisol concentration below the lower normal limit for the testing method in at least one of the three time points, 44 (54.32%) were above the upper limit of the laboratory norm, and in 9 (11.11%) we observed alarmingly high cortisol levels exceeding 36.2 μg/dL.

Within the observation period, cortisol concentration dropped over time regardless of the GC use. In the NGCG, cortisol concentration was significantly higher in each of the three time points as compared with the GCG (*p* << 0.0001); the same rule applied to ACTH (*p* << 0.0001). The ACTH concentrations differed between the GCG and NGCG (*p* << 0.0001). The detailed results are summarized in Table 1, Table 2 and Table 3.

**Figure 2:**
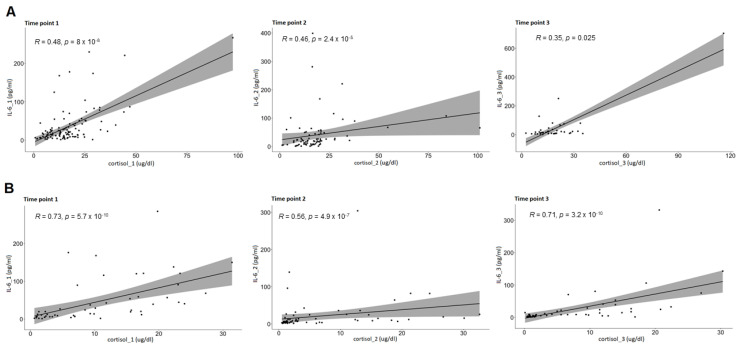
The correlations between cortisol and IL-6 levels throughout the three time points. (**A**) The NGCG, (**B**) the GCG.

In each group, cortisol and ACTH were correlated with a range of inflammatory markers: IL-6, CRP, leukocytes, neutrocytes, and lymphocytes. In both the GCG and NGCG, positive correlation was found between cortisol and IL-6 concentrations in all three time points (the GCG—cortisol_1:IL-6_1: *p* << 0.0001; cortisol_2:IL-6_2: *p* << 0.0001; cortisol_3:IL-6_3: *p* << 0.0001; the NGCG—cortisol_1:IL-6_1: *p* << 0.0001; cortisol_2:IL-6_2: *p* << 0.0001; cortisol_3:IL-6_3: *p* = 0.025). In the GCG, positive correlation between cortisol and CRP levels was found in all three time points (cortisol_1:CRP_1: *p*= 0.006; cortisol_2:CRP_2: *p*= 0.015; cortisol_3:CRP_3: *p* << 0.0001). Additional correlations between cortisol and other markers of inflammation were found, however they were not consistent throughout all three time points. The laboratory results are displayed in detail in Table 1, Table 2 and Table 3, and correlations between cortisol and IL-6 levels throughout all three time points in Figure 2.

The NGCG and the GCG were assessed in terms of endpoint completion. We checked, separately for the NGCG and the GCG, if cortisol measured at point #1 (cortisol_1) can be seen as an independent predictor of endpoint completion. In the next step, we wanted to assess if there was a cortisol level cutoff above which general chance of endpoint occurrence becomes significant. The logistic regression analysis showed that cortisol_1 can be seen as an independent predictor of meeting the primary endpoint in the NGCG (*p* < 0.05, both with and without confounders). More so, every increase in cortisol concentration by 1 μg/dL made the endpoint occurrence 13% more likely. In the GCG, cortisol_1 could not be seen as an independent predictor for endpoints.

For the NGCG, the optimal predictive cutoff value for endpoint occurrence for cortisol_1 was 15.45 μg/dL with area under curve (AUC) of 0.73, sensitivity of 75%, and specificity of 69%. Using this model statistically significantly improved the odds of correct classification of patients into the “endpoint” vs. “no endpoint” groups (OR = 6.7 [2.1–21.3], *p* < 0.005). To further verify the model’s usefulness, it was used on a test group, giving back 79% correct classifications (15 out of 19). Patients with cortisol_1 exceeding 15.45 μg/dL had a significantly higher odds ratio for meeting the primary endpoint (OR = 11.0 [1.1–106.4], *p* < 0.05). Introduction of the confounding factors to the model (age, sex and BMI) improved the performance on the training set (0.88 AUC, sensitivity of 96% and specificity of 84%), however it did not lead to the improved performance on the test set (60% accuracy).

For the GCG, the optimal predictive cutoff value for endpoint occurrence for cortisol_1 was 5.3 μg/dL with area under curve (AUC) of 0.72, sensitivity of 86.4%, and specificity of 63.6%. Using this model statistically significantly improves the odds of correct classification of patients into the “endpoint” vs. “no endpoint” group (OR = 11.3 [3.4–37.9], *p* < 0.005). To further verify the model’s usefulness, it was used on a test group, giving back 55% correct classifications (12 out of 22). The introduction of the confounding factors (age, sex and BMI) slightly improved the model performance on the training set (0.82 AUC, sensitivity of 83% and specificity of 73%) and the test set (70% accuracy).

In summary, due to low and intermediate levels of classification accuracy in the test groups (55% and 79%) and rather low specificity, cortisol_1 can be viewed as an independent predictor of endpoints only in the NGCG, optimally with the confounders being considered.

**Figure 3:**
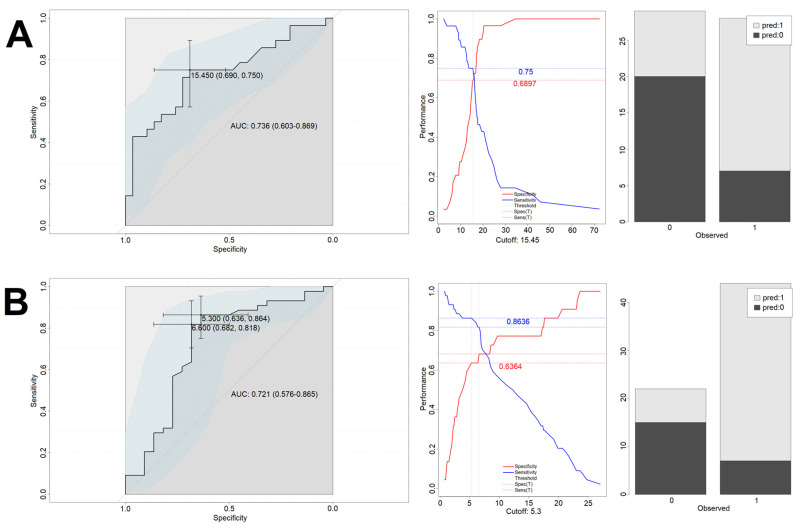
Performance of the cortisol-only based model on the training set. Left column depicts the ROC curves of the logistic regression models. Middle column presents analysis of balance between sensitivity and specificity, which was used to determine the optimal cut-off level. Right column shows the accuracy of predictions on the training set based on the determined cut-off. *X*-axis describes the real outcomes. Note the two possible cut-off levels in the bottom left figure. Both yielded the same Youden index. In the further analysis, the first one was used. Colors describe the predicted outcomes. 0—Lack of endpoint fulfillment; 1—endpoint fulfillment. *Y*-axis depicts counts. (**A**) NGCG group. (**B**) GCG group.

**Figure 4:**
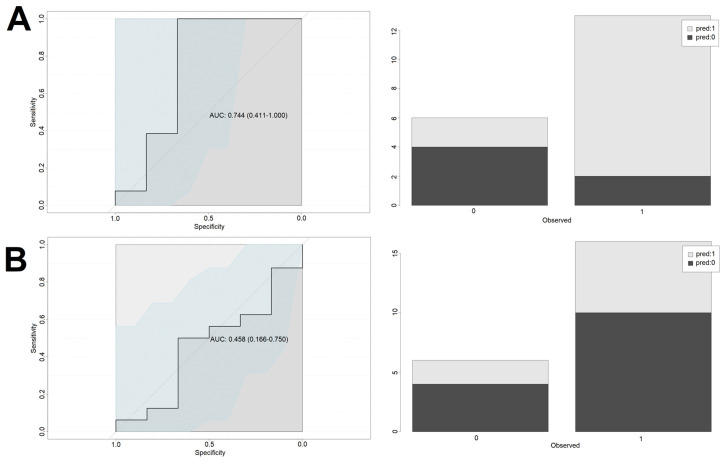
Performance of the cortisol-only based model on the test set. Left column depicts the ROC curves of the logistic regression models. Right column shows the accuracy of predictions on the test set based on the cut-off determined using the training set. *X*-axis describes the real outcomes. Colors describe the predicted outcomes. 0—Lack of endpoint fulfillment; 1—endpoint fulfillment; *Y*-axis depicts counts. (**A**) NGCG group. (**B**) GCG group.

**Figure 5:**
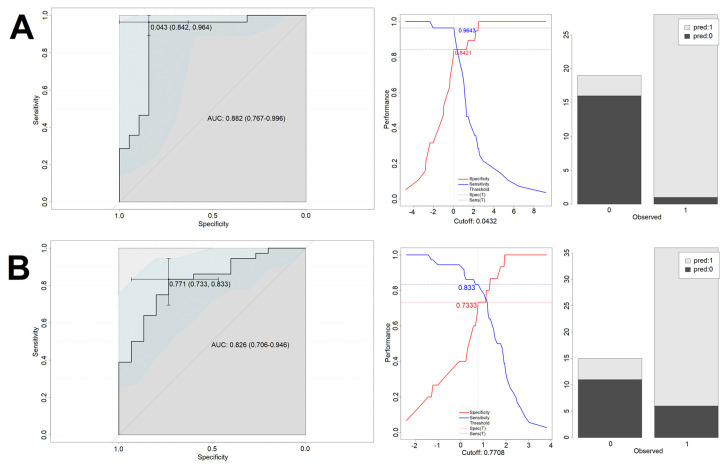
Performance of the cortisol-based model with confounding factors on the training set. Left column depicts the ROC curves of the logistic regression models. Middle column presents analysis of balance between sensitivity and specificity, which was used to determine the optimal cut-off level. Right column shows the accuracy of predictions on the training set based on the determined cut-off. *X*-axis describes the real outcomes. Colors describe the predicted outcomes. *Y*-axis depicts counts. 0—Lack of endpoint fulfillment; 1—endpoint fulfillment. (**A**) NGCG group. (**B**) GCG group.

**Figure 6:**
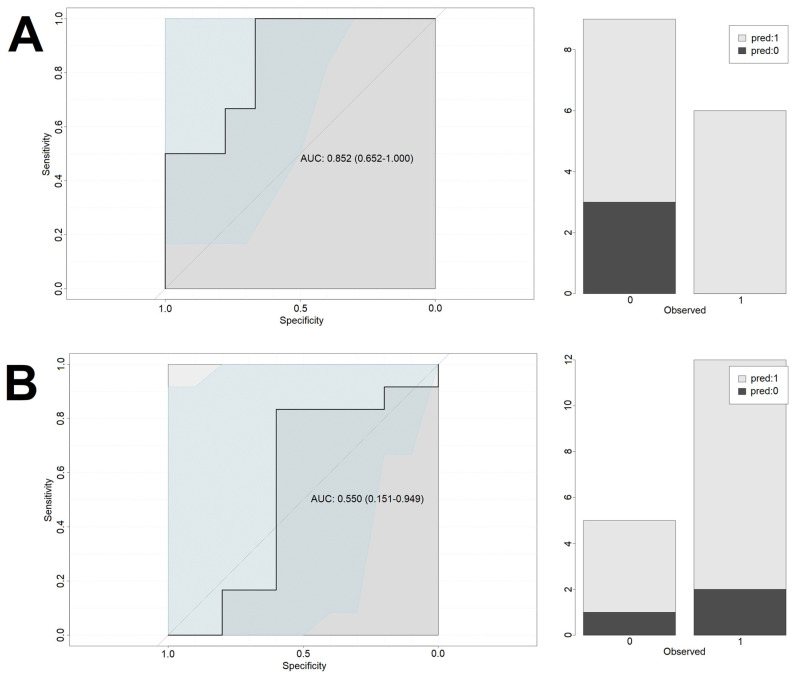
Performance of the cortisol-based model with confounding factors on the test set. Left column depicts the ROC curves of the logistic regression models. Right column shows the accuracy of predictions on the test set based on the cut-off determined using the training set. *X*-axis describes the real outcomes. Colors describe the predicted outcomes. *Y*-axis depicts counts. 0—Lack of endpoint fulfillment; 1—endpoint fulfillment. (**A**) NGCG group. (**B**) GCG group.

Performance of the models is presented in Figure 3 (cortisol-only model—training set), Figure 4 (cortisol-only model—test set), Figure 5 (model with additional confounding factors—training set), and Figure 6 (model with additional confounding factors—test set).

To further understand the observed problem, we compared cortisol levels in patients who met the primary endpoint and in those who did not. The analysis showed that cortisol_1 concentration was significantly greater in the NGCG subjects who met the primary endpoint than in those who did not (*p* << 0.0001). In the NGCG, the mean cortisol_1 level equaled 19.774 ± 13.737 μg/dL in individuals who met the endpoint (range 0.7–97.4 μg/dL) and 12.777 ± 7.554 μg/dL (range 1.6–33.1 μg/dL) in the no-endpoint group. In the GCG, there was no statistically relevant difference between the endpoint and no-endpoint groups.

Among secondary endpoints, cortisol_1 concentration could be seen as a significant predictor of endpoint occurrence in patients who died and/or used vasopressors, and this rule applied only to the NGCG. There was no statistically significant relationship between cortisol_1 and other secondary endpoints neither in the NGCG nor the GCG.

## 4. Discussion

To the best of our knowledge, this research is the first offering detailed assessment and comparison of adrenal function in two separate groups of COVID-19 patients: those receiving and not receiving GCs. So far, available articles excluded patients using GCs, therefore the data we collected shed new light on the topic [2,3,4,5]. Our approach involved evaluation over three separate time points with special regard for the initial test results obtained within the first 24 h of hospitalization. We focused on early post-admission hormonal screening to see whether it could be perceived as a reliable prognostic factor for detrimental in-hospital events such as death, mechanical or non-invasive ventilation, vasopressor use, and prolonged hospital stay, which were all defined as endpoints of this study. We consider the rarity and novelty of the presented data, consistent materials and methods promoting a unified study environment for all suitable patients, inclusion of both the NGCG and the GCG, and size of the studied sample (as compared with other projects) to be key strengths of our research. However, we must admit that our project had its limitations, such as no follow-up after hospitalization, single study site, and fixed laboratory norms different in the GCG and the NGCG.

Once the COVID-19 pandemic spread worldwide, scientists and physicians started seeking potential salvage therapies. The RECOVERY trial proved the efficacy of synthetic GC dexamethasone in improvement of survival in COVID-19 patients requiring oxygen supplementation [6]. Additional studies proved the beneficial effect, as well as efficacy of different GCs, such as methylprednisolone or hydrocortisone, in a similar setting [7,8,9,10]. Since that time, GCs have become a staple of care in COVID-19-derived respiratory failure and are continuously recommended by the WHO in patients requiring supplemental oxygen [11,12].

The HPA axis is essential for survival as it promotes the “flee or fight” response. GCs maintain the energetic homeostasis of the human body, as well as support its hemodynamic stability, promote growth and fertility, modulate immunity, and manage many other physiological functions [13,14]. Short-term hypercortisolemia can be beneficial in terms of survival, nevertheless prolonged exposure to elevated circulating steroids can be detrimental and provoke a number of, often serious, complications [15].

Exogenous steroids evoke potent biological action by providing potent negative feedback to the HPA axis. As it is commonly known, even 1 mg of synthetic steroid dexamethasone can suppress cortisol production overnight, a property which is used as a physiological background for the overnight low-dose dexamethasone suppression test. However, certain conditions, such as for example severe illness, active alcoholism, or pregnancy, can trigger excess cortisol production and non-responsiveness to the normal physiological stimuli known as non-neoplastic hypercortisolemia [16,17,18]. Rare conditions, such as, for example, intermittent hypercortisolemia, can result in surges of cortisol which can be difficult to capture in standard evaluation [19]. In the GCG group, full suppression of cortisol secretion in any of the three time points was not as widespread as one might expect despite the supraphysiological doses of administered glucocorticoids. This finding clearly points at COVID-19 as an excessive biological stressor which disturbs the homeostasis of the HPA. In our material, we encountered extreme IL-6 levels, which were as high as up to 10,000 pg/mL, and IL-6 can be distinguished as one of main components of exaggerated inflammation and a trigger of ACTH-independent cortisol production [4,20,21,22].

Multiple studies have shown that robust circulating cortisol in severe illness can be generally associated with poorer prognosis and oftentimes is a predecessor of death [23,24,25,26,27]. Our data confirmed that cortisol in the NGCG can be seen as an independent prognostic factor of hospitalization outcomes. The higher the initial cortisol, the higher the likelihood of undesired events, and a cortisol level increase by 1 μg/dL brings a 13% more likelihood of their manifestation. The analysis showed that cut-off value of 15.45 μg/dL in the NGCG can be independently tied with meeting the unfavorable endpoints. In our opinion, this may be due to the advanced stage of the disease at admission as cortisol concentration was closely tied with levels of circulating inflammatory markers. Increased cortisol levels accompanied high levels of IL-6 both in the GCG and the NGCG, as well as elevated CRP in the GCG. Data on the prognostic value of cortisol concentration in COVID-19 remain limited and evidence is scarce [2,3,5,27,28,29]. However, the available studies tend to point out that abnormally high circulating cortisol accompanies the severe form of the disease and predicts increased mortality, which is further supported by our own data.

Our research presented a profile of hormonal and inflammatory markers both in the NGCG and GCG and proved that cortisol concentration is associated with degree of generalized inflammation. Similar data were recently reported in a detailed assessment of the relationship between the HPA and inflammatory markers [4]. As increased IL-6 can be seen as one of the indicators of potentially deadly cytokine storm, it seems natural for the body to respond via HPA axis hyperactivation, resulting in a surge of anti-inflammatory mediators [20]. More so, it has been suggested that IL-6 may act as a co-factor of cortisol secretion, partly taking up the role of ACTH [4]. Indeed, in the course of a severe illness, high cortisol levels can be maintained even without the conventional positive feedback generated by ACTH. It has been implied that adipokines, proinflammatory cytokines, vasoactive agents, and bacterial toxins can provoke cortisol production. At the same time, cortisol breakdown and clearance in kidneys and liver is impaired due to insufficient activity of metabolizing enzymes [21,22]. As a result, cortisol concentration can remain elevated even despite the possible decrease in adequate central signaling.

The important role of cortisol in COVID-19 might not be limited to the acute phase of the disease. Long COVID syndrome is an attention-gaining result of COVID-19 characterized by signs and symptoms of the disease persisting beyond the acute infection [30]. Interestingly, some of the proposed explanations of this phenomenon include reduced adrenal reserve and/or development of adrenal insufficiency [31,32,33]. This can be, at least to some extent, explained either by the destruction of the adrenal glands (primary adrenal insufficiency), or the inhibitory effect of glucocorticoids used as anti-COVID-19 medications on the pituitary (secondary adrenal insufficiency) [15,31,33,34]. Whenever there is a clinical suspicion of adrenal insufficiency, the patient should undergo a swift work-up to confirm or exclude the diagnosis [35].

The results of our research highlight the utility of basic hormonal tests as biomarkers of poor prognosis in COVID-19. We suggest adding cortisol to the baseline assessment at admission as it might predict unfavorable outcomes early during hospital stay. Morning cortisol should be assessed on the first day of hospitalization, and then repeated whenever necessary. High concentrations of cortisol carry considerable predictive value of meeting the selected endpoints, especially in patients who are not treated with steroids, and a cortisol concentration of 15.45 μg/dL seems to be the optimal cutoff value predicting the endpoint completion.

## 5. Conclusions

COVID-19 is worldwide problem still calling for urgent attention. Most patients display non-severe signs and symptoms, but some require urgent hospital stays to save their lives. Our research shows that there is a strong positive correlation between stress hormone cortisol and inflammatory markers. Cortisol measured at hospital admission can provide early warnings about possible unfavorable outcomes, including death, in patients not treated with steroids.

## Figures and Tables

**Table 1:** Characteristics of the NGCG and the GCG at the time point #1.

	NGCG						GCG					
Characteristics	N	Mean ± SD	LQ	Medium	UQ	Range	N	Mean ± SD	LQ	Medium	UQ	Range
Age (years)	118	67.229 ± 15.134	58.000	69.000	79.000	18.00–94.00	56	65.804 ± 14.984	56.750	68.000	75.750	31.00–90.00
BMI (kg/m^2^)	95	26.815 ± 5.146	23.620	25.760	29.530	17.33–40.89	44	29.213 ± 5.156	25.620	27.895	31.328	19.53–44.08
Oxygen demand (L/min.)	117	2.517 ± 5.237	0.000	0.000	4.000	0.00–30.00	56	10.562 ± 15.958	3.000	6.000	10.000	0.00–75.00
Hospital stay (days)	118	12.890 ± 9.135	7.000	10.000	18.000	1.00–50.00	56	10.429 ± 6.126	6.000	9.000	14.250	1.00–30.00
Cortisol (µg/dL)	111	17.000 ± 12.139	9.150	15.200	20.950	0.70–97.40	53	10.421 ± 8.249	2.600	9.300	16.800	0.60–31.40
ACTH (pg/mL)	109	23.999 ± 20.239	11.600	18.100	31.700	1.20–127.00	52	11.573 ± 19.735	1.200	2.265	13.850	1.20–95.80
IL-6 (pg/mL)	111	35.888 ± 47.938	9.390	19.400	41.650	1.410–267.00	53	233.631 ± 1368.543	7.210	20.000	59.100	1.41–10,000.00
CRP (mg/L)	112	61.400 ± 67.871	9.875	39.700	84.775	0.700–320.10	53	103.50 ± 100.612	21.700	78.100	139.900	1.00–427.70
Leukocytes (^10^3^/µL)	112	6.786 ± 3.578	4.375	5.845	8.152	1.790–26.16	53	6.706 ± 3.823	3.920	5.590	8.280	1.90–21.22
Neutrocytes (^10^3^/µL)	112	4.640 ± 3.241	2.570	3.560	5.610	1.220–21.36	53	5.29 ± 3.670	3.120	4.390	6.520	1.08–20.24
Lymphocytes (^10^3^/µL)	112	1.332 ± 0.652	0.858	1.260	1.755	0.260–3.56	53	0.898 ± 0.499	0.650	0.800	1.030	0.17–2.78

**Table 2:** Characteristics of the NGCG and the GCG at the time point #2.

	NGCG						GCG					
Characteristics	N	Mean ± SD	LQ	Medium	UQ	Range	N	Mean ± SD	LQ	Medium	UQ	Range
Age (years)	83	68.157 ± 14.374	60.000	70.000	78.500	18.00–94.00	74	65.459 ± 15.664	56.000	67.000	78.000	31.00–94.00
BMI (kg/m^2^)	67	26.533 ± 4.877	23.775	25.950	28.360	17.40–40.82	58	28.752 ± 5.501	24.828	27.815	31.250	17.33–44.08
Oxygen demand (L/min.)	82	2.293 ± 6.787	0.000	0.000	0.000	0.00–40.00	73	8.493 ± 11.168	0.000	5.000	11.000	0.00–60.00
Hospital stay (days)	83	13.458 ± 9.793	7.000	10.000	18.500	2.00–50.00	74	12.730 ± 5.836	8.000	12.000	16.000	4.00–30.00
Cortisol (µg/dL)	78	18.836 ± 14.801	11.900	16.600	20.475	1.20–101.00	70	6.369 ± 7.972	1.200	2.400	9.025	0.50–32.6
ACTH (pg/mL)	76	29.719 ± 29.915	13.500	20.200	38.400	1.20–216.00	69	9.602 ± 18.058	1.200	2.010	6.790	1.20–92.50
IL-6 (pg/mL)	78	41.296 ± 62.231	8.975	21.000	48.275	1.41–399.00	70	39.765 ± 167.153	3.990	7.920	18.100	1.41–1374.00
CRP (mg/L)	79	52.347 ± 60.984	9.150	36.300	66.000	0.70–281.80	70	40.333 ± 41.274	13.875	24.750	57.425	0.80–171.30
Leukocytes (^10^3^/µL)	80	6.576 ± 3.230	4.355	5.615	8.035	0.56–18.25	69	7.460 ± 2.861	5.200	7.500	10.090	2.39–13.32
Neutrocytes (^10^3^/µL)	80	4.392 ± 2.997	2.375	3.250	5.862	0.25–14.31	69	5.658 ± 2.522	3.450	5.880	7.270	1.33–11.66
Lymphocytes (^10^3^/µL)	80	1.331 ± 0.676	0.880	1.200	1.765	0.12–3.92	69	1.040 ± 0.585	0.670	0.920	1.230	0.35–3.42

**Table 3:** Characteristics of the NGCG and the GCG at the time point #3.

	NGCG						GCG					
Characteristics	N	Mean ± SD	LQ	Medium	UQ	Range	N	Mean ± SD	LQ	Medium	UQ	Range
Age (years)	46	68.435 ± 14.842	61.500	70.000	75.000	18.00–94.00	63	68.365 ± 14.544	61.500	69.000	80.000	31.00–94.00
BMI (kg/m^2^)	38	26.054 ± 4.267	22.868	25.735	28.263	19.10–38.30	49	29.302 ± 5.980	24.690	27.940	32.030	17.33–44.08
Oxygen demand (L/min.)	41	1.976 ± 5.303	0.000	0.000	0.000	0.00–30.00	61	4.984 ± 9.856	0.000	0.000	6.000	0.00–52.00
Hospital stay (days)	46	15.565 ± 8.400	9.000	13.500	20.000	4.00–41.00	63	15.952 ± 8.261	10.000	14.000	19.000	6.00–50.00
Cortisol (µg/dL)	40	19.925 ± 17.383	11.500	17.400	22.175	2.40–116.00	61	6.985 ± 7.525	1.000	3.100	11.900	0.10–30.30
ACTH (pg/mL)	40	32.039 ± 24.176	16.950	23.600	42.150	2.95–114.00	61	13.086 ± 20.446	1.220	2.890	22.200	1.22–107.00
IL-6 (pg/mL)	40	47.343 ± 115.409	8.705	16.900	29.275	1.41–704.00	61	140.251 ± 917.906	3.530	8.130	22.200	1.41–7182.00
CRP (mg/L)	40	47.150 ± 54.222	8.550	32.800	58.500	0.80–280.30	62	37.098 ± 67.678	3.450	7.900	30.700	0.80–342.60
Leukocytes (^10^3^/µL)	40	6.558 ± 2.678	4.920	5.770	7.195	1.92–16.96	62	10.039 ± 5.770	6.478	9.230	11.925	3.09–39.01
Neutrocytes (^10^3^/µL)	40	4.224 ± 2.734	2.638	3.385	4.970	0.68–15.84	62	7.702 ± 5.548	4.395	6.585	9.047	1.36–35.97
Lymphocytes (^10^3^/µL)	40	1.352 ± 0.621	0.865	1.290	1.768	0.43–3.19	62	1.287 ± 0.756	0.747	1.185	1.585	0.28–4.46

## Data Availability

The data can be made available upon reasonable request. Please contact Agata Berlińska, agata.berlinska@gumed.edu.pl.

## Supplementary Materials

The following supporting information can be downloaded at: https://www.mdpi.com/article/10.3390/biomedicines10071527/s1, Table S1: Laboratory norms and methods.
