# Supplementary material for: Cortisol as an Independent Predictor of Unfavorable Outcomes in Hospitalized COVID-19 Patients
Source: Biomedicines. 2022 Jun 28;10(7):1527. doi: 10.3390/biomedicines10071527 (PMC9313159; doi:10.3390/biomedicines10071527)
Supplement: Supplementary file 1 [file biomedicines-10-01527-s001.zip › biomedicines-1775911-supplementary.pdf]

Table S1: Laboratory norms and methods.

	Parameter	Reference norm with units	Laboratory	Analytical method	Analyzer
1.	ACTH	7.20-63.30 pg/ml	Diagnostyka Laboratoria, Gdańsk, Poland	Electrochemiluminescence assay	Cobas 8000, Roche
2.	cortisol	6.02-18.4 μg/dl (6:00-10:00 AM)	Diagnostyka Laboratoria, Gdańsk, Poland	Electrochemiluminescence assay	Cobas 8000, Roche
3.	CRP	0-5 mg/l	Diagnostyka Laboratoria, Gdańsk, Poland	Immunoturbidimetric assay	Cobas 6000, Roche
4.	IL-6	<5.9 pg/ml	Central Clinical Laboratory, University Clinical Centre, Gdańsk, Poland	Chemiluminescence immunoassay	Immulite XP, Siemens
5.	leukocytes	females: 3.98-10.04x10^3/μl<br>males: 4.23-9.07x10^3/μl	Diagnostyka Laboratoria, Gdańsk, Poland	Fluorescence flow cytometry	XT-4000i, Sysmex
6.	neutrocytes	2-7x10^3/μl	Diagnostyka Laboratoria, Gdańsk, Poland	Fluorescence flow cytometry	XT-4000i, Sysmex
7.	lymphocytes	1-3x10^3/μl	Diagnostyka Laboratoria, Gdańsk, Poland	Fluorescence flow cytometry	XT-4000i, Sysmex
